# Shared decision making in musculoskeletal pain consultations in low- and middle-income countries: a systematic review

**DOI:** 10.1093/inthealth/ihz077

**Published:** 2019-11-15

**Authors:** Sreya Sam, Radha Sharma, Nadia Corp, Chinonso Igwesi-Chidobe, Opeyemi O Babatunde

**Affiliations:** University School of Medicine, Keele University, Staffordshire ST5 5BG, UK; University School of Medicine, Keele University, Staffordshire ST5 5BG, UK; Arthritis Research UK Primary Care Centre, Research Institute for Primary Care & Health Sciences, Keele University, Staffordshire ST5 5BG, UK; Arthritis Research UK Primary Care Centre, Research Institute for Primary Care & Health Sciences, Keele University, Staffordshire ST5 5BG, UK; Arthritis Research UK Primary Care Centre, Research Institute for Primary Care & Health Sciences, Keele University, Staffordshire ST5 5BG, UK

**Keywords:** global health, musculoskeletal pain, patient-centred care, quality of care, shared decision making

## Abstract

**Background:**

Global populations, especially those in low- and middle-income countries (LMICs), are at an increased risk of musculoskeletal (MSK) pain, a leading cause of years lived with disability. Shared decision making (SDM) in the management of these conditions may drive improvements in healthcare outcomes and quality. This study aimed to synthesize and appraise available evidence regarding SDM in MSK pain consultations in LMICs.

**Methods:**

Comprehensive literature searches were conducted in 12 databases for primary studies investigating SDM in MSK pain consultations across all healthcare and community settings in LMICs. Study eligibility screening, data extraction and quality appraisal (using the Critical Appraisals Skills Programme tool) were completed by pairs of reviewers. Findings were brought together using thematic synthesis of data from all the primary studies.

**Results:**

Seven studies (mostly moderate quality) were included. There was low awareness of SDM among healthcare professionals (HCPs); however, this is not explicitly practised due to cultural and operational barriers. HCP training and patient empowerment through health literacy were proposed facilitators. The traditional paternalistic approach to treatment poses a key barrier to SDM, decreases adherence to prescribed treatments and raises the risk of poor clinical outcomes.

**Conclusions:**

SDM is still a relatively ‘foreign concept’ within consultations and management of MSK pain patients in LMICs. There is a dearth of research in SDM and patient-centred care. Given the socio-economic impact of MSK pain, further research into the value of SDM in LMIC healthcare settings requires further consideration.

## Introduction

Globally, musculoskeletal (MSK) pain conditions account for 21.3% of total years lived with disability as a result of chronic pain and decreased functionality.^1,2^ The socio-economic and health impacts of MSK pain conditions are particularly significant in low- and middle-income countries (LMICs), as most of the workforce, and >60% of the adult population, are at increased risk of developing MSK pain.^3,4^ In terms of management, these conditions can be complex, require long-term management and increase pressure on healthcare services, yet the outcomes of care vary widely.^5,6^ In LMICs, outcomes are often particularly poor, increasing the risk of absenteeism, low productivity and resultant loss of an active workforce.^7–9^

Consultations involving shared decision making (SDM), with the aim of agreement in management plans between doctors and patients, have been widely regarded as good practice.^10^ This informs patients about available options, such that patients can assess the costs, risks, benefits and concordance of the different treatments with their personal beliefs and needs, under the guidance of trained healthcare professional (HCPs).^11,12^ This form of consultation has shown positive effects in high-income countries (HICs), allowing the patient to be at the forefront of care, within a mutualistic HCP–patient relationship.^13–15^ Furthermore, SDM approaches to patient education and advice for self-management and prevention of further disability have been shown to be key determinants of care for MSK pain in HICs.^6,10^

The global reach of SDM is increasing through literature and advocacy for patient-centred care, and research on the impact of SDM on MSK pain management is evolving.^10,12–14^ Due to patient empowerment, and more appropriate ownership of one’s own care, SDM may be critical to optimizing outcomes of care, health-related quality of life (QOL) and appropriation of limited healthcare resources in LMICs.^16^ Previous systematic reviews have employed methodologically robust evidence synthesis to explore the effectiveness of SDM interventions and barriers and facilitators of SDM in clinical practice,^17–19^ and found SDM to be positively associated with improved patient satisfaction, treatment adherence and symptoms reduction, although estimates of the effects were small and not always statistically significant.^17–19^ However, evidence from these bodies of literature were underpinned by data from HICs with well-established and thriving healthcare systems. SDM research and practices within healthcare systems in HICs have also progressed across condition- and population-specific contexts.^20,21^ Despite the increased risk and the attributed health and socio-economic burdens of living with MSK pain in LMICs, it is not known to what extent there is awareness, use and practice of SDM in the management of MSK pain in these settings. Furthermore, an understanding of the factors that may influence the practice of SDM in LMICs, within the context of MSK pain conditions, are yet to be systematically explored. This study aimed to summarize and appraise available evidence regarding SDM in MSK pain management in LMICs. The specific objectives were to explore the awareness of and use of SDM in MSK pain consultations in LMICs, explore patient-oriented outcomes of care associated with SDM in MSK pain consultations in LMICs and identify the barriers and facilitators of SDM in the management of MSK pain in LMICs from both the patient and HCP perspectives.

## Methods

### Protocol and registration

A protocol was developed a priori for this systematic review and was registered with the international prospective register of systematic reviews—PROSPERO (registration number CRD42018096052). This systematic review has been conducted and reported according to Preferred Reporting Items for Systematic Reviews and Meta-Analyses (PRISMA) guidelines.^22^

### Eligibility criteria

Studies were included if they involved adults ≥18 y of age with diagnostic or symptomatic MSK pain and/or HCPs managing these conditions (e.g. physiotherapists, nurses, occupational therapists, general practitioner equivalents certified in the care of patients). Studies were excluded if participants had ‘red flag’ conditions that would be indicative of a more serious underlying pathology other than MSK pain problems (e.g. suspected fractures and recent trauma, cancer, inflammatory arthritis, pregnancy-related pain)^23^ or were vulnerable patients who had cognitive impairment, dementia or terminal illness. Studies were also included if they included a discussion of management options with a patient by an HCP (including surgical management, prescribed and over-the-counter medications, physiotherapy, occupational therapy and alternative therapies), patients’ and/or HCPs’ perceptions towards shared decision making, perceived barriers and facilitators to SDM from patients and HCPs, patient-related clinical outcomes (pain, disability/function and health-related QOL) and/or health utilization-related outcomes (efficiency of care and frequency of consultation). Full-text publications, conference reports and protocols of ongoing studies were eligible if they reported results relating to any of the stated review outcomes. Any primary study design, with the exception of case studies across all healthcare (primary, secondary and tertiary care) and community settings of LMICs, was eligible for inclusion. LMICs were defined as those with a gross national income per capita of ≤US$1005 (low income) and those between US$1006 and US$3955 (low middle-income) in 2016.^24^

### Information sources and searches

A comprehensive search strategy including terms related to shared decision making, MSK conditions and LMICs according to World Bank classifications was developed (see Appendix 1 for the detailed MEDLINE search strategy). MEDLINE, Embase, CINAHL Plus, PsycINFO, AMED, Global Index Medicus, Cochrane Central Register of Controlled Trials, Web of Science and PEDro electronic databases were searched from inception until 1 June 2018. Grey literature databases were also searched (Open Grey, NDLTD: Networked Digital Library of Theses and Dissertations [Global ETD search] and the WHO Institutional Repository for Information Sharing [IRIS]). In addition, reference checking and citation tracking of eligible articles were conducted. There were no restrictions to date of research or language of publication.

### Study selection

Titles and abstracts of studies were screened using Covidence (systematic review software; www.covidence.org) by pairs of independent reviewers (SS, RS, OOB, NC) based on the eligibility criteria (see Box 1). Full texts of relevant studies were subsequently assessed for eligibility using the same selection process as the titles and abstracts. Any disagreements regarding eligibility at each stage of the selection process were resolved through discussion between pairs of reviewers or by consensus in research team meetings.

### Data collection process and data items

The studies that met the required criteria underwent data extraction and quality appraisal. Independent double data extraction was performed using a data collection pro forma (in Excel [Microsoft, Redmond, WA, USA]) that was designed, piloted and tested by the research team prior to data extraction. Extracted data included study population demographics (including comorbidities and MSK diagnosis, profession and level of training/practice for HCPs), consultation/intervention characteristics (content, HCP–patient relationship), clinical outcomes (pain, function/disability and health-related QOL), barriers and facilitators of SDM in consultation/management, and HCP and patient attitudes to patient-centred care.

### Methodological quality appraisal of included studies and grading of evidence

Methodological quality of included studies was assessed by paired independent reviewers (RS, SS, OOB, NC) using the Critical Appraisal Skills Programme tools.^25^ Within this, the appropriateness of each study’s research design, recruitment strategy, data collection process and rigour of data analysis were considered and assessed as ‘yes’ when reported procedures satisfied the methodological criteria as stated in the tool or ‘no’ when it was clear criteria had not been met. Where there was not information to judge the appropriateness of aspects of study design and methodology, such were marked as ‘unclear’. Overall, where there had been consistent satisfaction of criteria across studies, such aspects were designated as having an overall low risk of bias. Conversely, a high risk of bias was assigned where standard methodological criteria had not been met. Conflicts regarding appraisal were discussed and resolved by consensus as a team. The overall grade of evidence from the review was determined using the Confidence in Evidence from Reviews (GRADE-CERQual) criteria.^26^ To assesses this, overall methodological limitations, relevance, coherence and adequacy of data were taken into consideration across studies.

### Data analysis

Thematic synthesis of data was conducted in three stages without a priori codes.^27^ Stages involved line-by-line coding of texts from the primary studies, the development of ‘descriptive themes’ across the included studies and the generation of analytical themes. Coding was conducted inductively by two independent reviewers (RS, SS). Where applicable, discrepancies in coding were resolved by discussion and consensus in a team meeting. This was followed by team discussion (OOB, RS, SS, NC, CI-C) of the data from included studies to identify, describe and aggregate consistent codes across studies while ensuring that the development of descriptive themes remained closely aligned with data from the primary studies. Emerging descriptive themes were then interpreted and analytical themes were identified. Analytical themes identified from key descriptive themes reflected patient and HCP perspectives in relation to proposed outcomes, as supported by data from the studies. Descriptive quantitative data (including counts and percentages) were extracted into tables and presented with study characteristics, and findings of the review across all three stated outcomes were reported in a narrative synthesis.

## Results

### Characteristics of included studies

**Figure 1:**
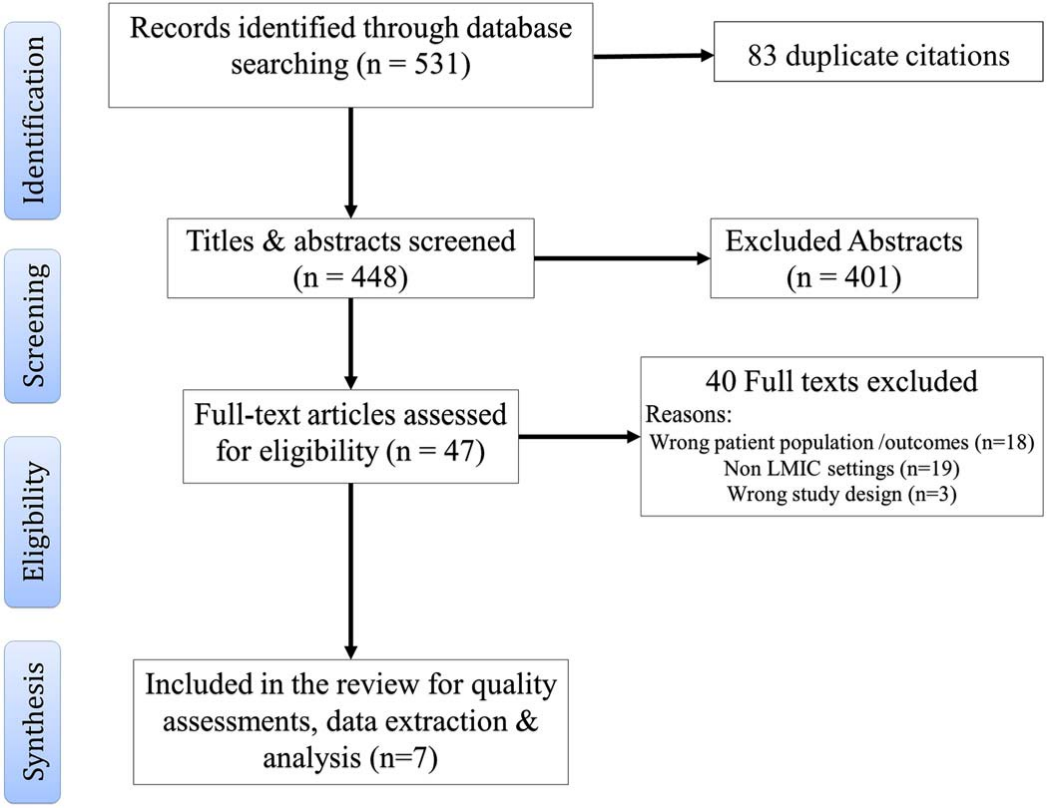
Study flow chart (PRISMA).

Of 466 citations, seven studies (one randomized clinical trial, six cross-sectional)^28–34^ met the eligibility criteria and were included in this review (Figure 1). No relevant studies were identified through additional searching of grey literature, references or citation tracking of included studies. The studies (published in the 5 y between 2012 and 2017) presented data regarding patient consultations for chronic, non-specific back pain, fibromyalgia, osteoarthritis and osteoporosis in middle-income countries (Iran, Romania, Mexico, Dominican Republic, Brazil and Russia). Where reported, patient participants were predominantly females, with ages ranging between 18 and 80 y. Settings of care for participants across included studies were mostly secondary (n=5) and tertiary/unclear (n=2). References to care and consultations were mostly in relation to publicly funded healthcare systems. Detailed characteristics of included studies are presented in Table 1.

**Table 1:** Characteristics of included articles

Author	Country	Study design	Setting of care	Participant characteristics	Sampling methods	Analysis design	MSK pain condition	Aim of the study	Main focus	HCP–patient relationship
Colmenares-Roa et al.^28^	Mexico	Qualitative: Ethnographic study –observations + interviews	Secondary and private	Patients: n=8; age 34–74 y; overall 37.5% male (public 25% male; private healthcare, 50% male) HCPs: rheumatologists (Mexican College of Rheumatology certified) with 6–27 y professional experience; n=4; 50% male	Purposive	Narrative analysis	^*^Fibromyalgia	To examine the doctor–patient relationship between patients with fibromyalgia and rheumatologists in Mexican private and public care settings	Barriers and facilitators	Paternalistic: public health Mutualistic and paternalistic: private health
Devine et al.^29^	Dominican Republic	Qualitative: semi-structured interviews	Tertiary	HCPs: surgeons and nurses, n=12 (2 orthopaedic, 1 general, 1 gynaecological surgeon, 2 medical interns, 4 nurses, 2 nurse assistants)	Purposive	Content analysis	Total joint replacement (postoperative pain management)	To explore differences in postoperative decision making between HCPs in America and the Dominican Republic	Barriers	Paternalistic
Rashidian et al.^31^	Iran	Qualitative: interviews	Secondary care	Patients: n=8; 37.5% male HCPs: physicians n=14 (3 endocrinologists, 2 general surgeons, 2 neurologists, 2 oncologists, 1 cardiologist, 1 dermatologist, 1 gastroenterologist, 1 ophthalmologist, 1 urologist)	Purposeful, maximum variation	Thematic analysis	Arthritis	To describe barriers and/or limitations, as well as benefits of using patient decision aids in Iran	Barriers and limitations	Paternalistic
Fagundes et al.^32^ (study protocol)	Brazil	RCT	Secondary care	Patients: n=222 estimated, between 18 and 80 y of age	Random, three arm	–	Chronic, non-specific back pain	To determine the efficacy of combining a therapeutic alliance with minimal intervention on outcomes of patients with chronic, non-specific low back pain	Facilitators	Unknown
Gasparik et al.^31^ (conference presentation notes)	Romania	Qualitative	Secondary care	HCPs: rheumatologists, n=3	–	Transversal analysis of consultations	Osteoporosis	To evaluate consultation structure and communication between rheumatologists and patients with osteoporosis	Barriers	Paternalistic
Zamanzadeh et al.^29^	Iran	Qualitative: unstructured and semi-structured interviews	Secondary care	Patients: n=17; age 39–75 y; 29.4% male HCPs: with ≥5 y of work experience with osteoarthritis, n=8	Purposeful	Content analysis	*Osteoarthritis	To explore patient experiences of osteoarthritis pain management in Iran	Barriers and facilitators	Consumeristic
Zakroyeva et al.^33^ (conference abstract)	Russian Federation	Cross-sectional survey	Unknown	Patients: n=128; mean age 64 y (95% CI 63 to 66); mostly women HPCs: doctors, n=22	Convenient	Descriptive	Osteoporosis	To assess patients’ awareness of their condition in comparison with doctors’ judgement of perceived patient knowledge	Barriers and facilitators	Unknown

*American Rheumatology Association criteria.

### Methodological quality of included studies

**Figure 2:**
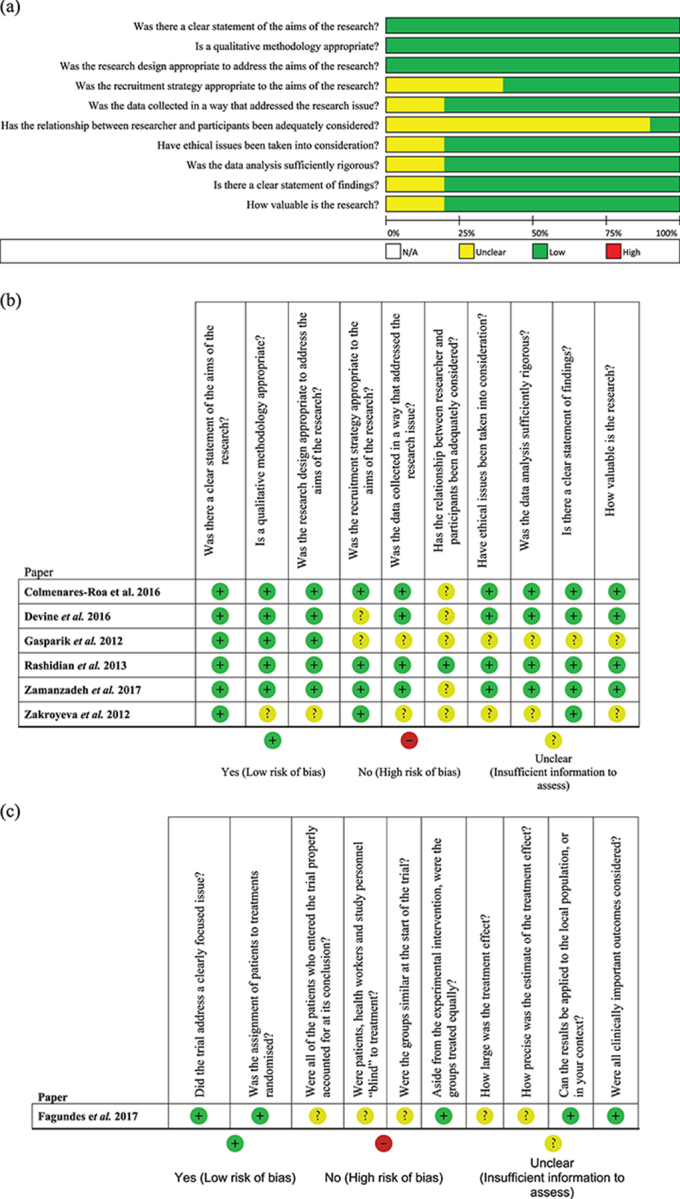
Methodological quality assessments. (a) Overall summary of quality assessments for all included studies. (b) Quality assessment of qualitative studies. (c) Quality assessment of the randomized clinical trial.

Outcomes of methodological assessments of each of the included studies are presented in Figure 2a–c. Only one study^31^ was assessed to have satisfied the criteria across quality domains and was judged to be of high methodological quality. All other studies were of moderate quality. However, many aspects of methodological quality of the included studies were assessed as unclear due to a lack of sufficient information to judge them as either having fully met and satisfied quality criteria or not (Figure 2c). Specifically, >80% of the studies did not adequately explore the relationship between the study researchers and participants and were judged as unclear. In reality, it is possible that this important source of bias was not addressed in many of the study designs. The clear statement of study questions within each report and the justification of qualitative methodology in addressing each study’s aims equivocally satisfied the quality assessment criteria and were judged as having a low risk of bias across included studies. Some aspects of the methodological quality of the only randomized clinical trial^33^ included in this review could not be assessed due to it being a report of an ongoing study without full trial results (Figure 2b).

### Review outcome 1. Patient and HCP awareness of SDM for MSK pain consultations

Six studies^28–32,34^ (moderate quality) explored the perspectives of HCPs (n=63). Overall, there was limited evidence to support awareness, use or intention to practise SDM in MSK pain management by HCPs, as only two studies^28,33^ implied explicit awareness of SDM. Although many HCPs in these two studies reported awareness of elements of SDM and/or patient-centred care, there appears to be a perception–reality gap, as many of the reported perceptions of awareness could not be mapped on-to known SDM concepts. Six studies,^28,30–34^ mostly of moderate methodological quality, explored patients’ (n=383) experiences regarding SDM for MSK pain. Similarly, to HCPs, patient awareness of SDM as a specific term was not explicitly stated across studies, but in a generalized context. Patients’ views were expressed in relation to HCP–patient interactions, communications during consultation and understanding of the management of their MSK condition(s) as important values of good care.

### Review outcome 2. Associations between SDM in MSK pain consultations and patient outcomes

Studies primarily reported perceived clinical outcomes. Four studies^28,30,31,33^ explored the effect of specific interventions or consultation in the context of SDM on clinical outcomes of pain, function and QOL. Of these, none empirically evaluated the relationship between SDM and an explicitly measured patient outcome. Only one study^33^ explicitly mentioned discussion of pain management within consultations. In this and three other studies^28,30,31^ where patients reported receiving some form of SDM (essentially a discussion of the appropriateness of prescribed care), a positive and mutualistic HCP–patient relationship and SDM was perceived or expected to be related to lower pain levels due to improved acceptance, understanding of treatment goals and adherence to treatment. Where patients had no form of SDM,^29^^,^^32–34^ little or no improvement in pain and poor adherence to treatment(s) was reported. For instance, poor drug counselling perpetuated patient engagement with symptomatic use of prescriptions, curtailing effective resolution of pain and other symptoms.^29,30^

The lack of SDM in consultations was also perceived to result in compromised functional ability and health-related QOL. Patients described pain that was sometimes so severe they were unable to sleep,^30^ affecting their family life or daily living.^28,30^ Poor drug counselling (including poor discussion of the benefits and risks of prescribed medication options) implied that patients were unaware of the possible side effects of medications that might interfere with their daily living. Consequently, patients were unwilling to continue with prescriptions and often sought alternative practitioners for treatment and answers that HCPs were unable to provide. Patients also reported going through emotional stress, further accentuating poor functional and health-related QOL outcomes.^28,30^ Findings also identified patient impressions of HCPs as key to actual and/or expected outcomes of care.^28,30,31^

### Review outcome 3. Barriers and facilitators of SDM

Table 2 provides an overview of the main themes and subthemes presented from the perspectives of patient and HCPs. Key descriptive themes were grouped under two broad analytical themes: factors affecting awareness, evidence of use or practice of SDM in consultations and management of MSK pain conditions, and operationalization and impact of SDM (i.e. conceptualization of HCP–patient interaction in consultations and impact [outcome] of the use or non-use of SDM in MSK pain consultations on patients and HCP practice).

**Table 2:** Summary of findings on themes and subthemes (with references) using the CerQUAL criteria

Analytical themes	Cross- cutting	Descriptive themes and evidence base (refs)	Assessment of methodological limitations	Assessment of relevance	Assessment of coherence	Assessment of adequacy	Overall assessment of confidence	Comments/explanation of judgement
Awareness, use and practice of SDM	Patient perspective	Lack of awareness and practice^28,29,31,32,34^	Minor concerns as methodology of two studies^32,33^ could not be assessed in detail	Partial relevance, as some studies do not directly pose questions regarding awareness of SDM	No concerns, as patterns are consistent across studies	Some concerns	Moderate confidence	Graded as moderate confidence because of minor concerns regarding methodological limitations, relevance and adequacy of data
		Evidence of awareness and practice^30,32^						
	HCP perspective	Lack of awareness and practice^28–31^^,^^34^	Minor concerns regarding methodological limitations in two studies^29,34^	No concerns; evidence is well mapped	Some concerns due to findings of disparate data/viewpoints of HCPs within some studies	Minor concerns due to limited data	Moderate confidence	Graded moderate confidence because of concerns with methodology, coherence and limited data
		Evidence of awareness and practice^28,30,31^						
Conceptual barriers	Patient perspective	Perceived lack of empathy from HCP^28–32,34^	Minor concerns regarding methodological limitations in some studies ^29,32,34^	No concerns; evidence is well mapped	No concerns as patterns are consistent across studies	Minor concerns due to limited data in review (not within studies)	High confidence	Graded as high confidence as no concerns with relevance, coherence of available data
		Biomedical approach to patient management^28,30–32,34^						
		Perceived HCP skills^28,30–32,34^						
		Personal health beliefs^28,30,31,34^						
	HCP perspective	Infrastructure and resources • Consultation time and consultation spaces^28^^,^^30–32^ • Complexity of MSK condition^28^ • Increased patient caseload^28,29,31^	Minor concerns regarding methodological limitations in some studies^29,32,34^	No concerns; evidence is well mapped	No concerns as patterns are consistent across studies	No concerns; available data sufficiently support evidence	High confidence	Graded as high confidence as no concerns with relevance, coherence of available data
Operational barriers		• Unclear treatment pathway (clinical guidelines)^28,31^ • Available treatment options^28–31,34^ • Financial incentives^28,31^ • HCPs’ impression of patients, including patient labelling, minimization^28,31^ • Perceived patient limited awareness of SDM and health literacy^28–31,34^ • healthcare models and cultural factors^28–32,34^						
SDM facilitators	HCP perspective	Training in SDM^28–34^	Minor concerns regarding methodological limitations in some studies^29,32,34^	Partial relevance due to indirect exploration of this theme across studies	No concerns as patterns are consistent across studies	Moderate concerns due to limited data	Low confidence	Graded as low confidence due to concerns with methodology, relevance and thin data
		Patient decision aids^31^						
Other factors		Association between SDM in MSK pain consultations and patient outcomes • Pain^28–32,34^ • Function^30–32^ • Health-related quality of life^28–32,34^	Moderate concerns regarding collected data and analysis relating to this theme across studies	Substantial concerns about relevance, as studies did not explicitly measure patient-oriented outcomes of care	Minor concerns, as patterns were mostly (but not always) consistent across studies	Substantial concerns about adequacy because studies provided thin data regarding this theme	Very low confidence	Graded as very low confidence due to substantial concerns with methodology, relevance and adequacy of data

#### Barriers to SDM for MSK pain consultation and management (patient perspectives)

##### Awareness, use and practice of SDM

Across the six studies^28,30,31,33,34^ that explored patients’ experiences regarding MSK pain consultation and management, patients who consulted in publicly funded care settings were less likely to be health literate or be aware of SDM, suggesting that low healthcare affordability and low health literacy are barriers to patient awareness of SDM for MSK pain consultations.

##### Conceptualization of HCP–patient interactions

Factors such as a perceived lack of empathy, a biomedical approach to patient management, patient perception of HCP consultation and communication skills and personal health beliefs of patients were identified. Most studies (n=6) highlighted the role of empathy in MSK consultations.^28–32,34^ Some patients who received care in private settings suggested that treatment adherence and recovery relate to the empathy received from HCPs.^28,29,31^ Patients who received care in publicly funded care settings were more likely to report a lack of compassion or empathy from their HCPs during consultations and throughout their management.^28,29,31^ Patients reported delegitimization, including HCPs minimizing or denying the seriousness of their complaints,^28^^,^^30–32^ often resulting in perceived or actual amplification of symptoms in order to refute delegitimization.

Studies described that patients perceived a biomedical but superficial approach to care where, once ‘red-flag’ conditions had been ruled out, HCPs tended to slacken their investigations into the aetiology of patients’ symptoms,^28,29^ without an acknowledgement of the psychosocial aspects of patient illness. Such a ‘diagnostic limbo’ was reported by patients as terrifying.^28^

In some studies, researcher observations of consultations reported deficiencies of both illness history taking and physical examination of patients by HCPs. Patients also reported marked differences in HCP consultation and communication across public and private healthcare settings, with the latter showing more favourable dispositions.^28^ Additionally, the content and manner in which information was given in consultations were deemed insufficient, as patients reported neither having fully understood nor barely remembered what was said shortly after consultations.^28,34^ Consequently, patients were unaware of administration, adherence issues and potential side effects of the drugs they were given, as well as what to do if there was an adverse reaction. They also reported being unable to adapt the given information to self-manage their conditions.

Patients’ personal beliefs (e.g. perception of ‘patient-centred care’ from non-orthodox practices) influenced their healthcare-seeking behaviour and trust in HCPs.^28,30,31,34^ Also, opinions (e.g. regarding proficiency of physicians in particular care settings) of popular/lay referral sectors, such as important family members and friends who may be assisting patients in the interpretation of their pain conditions and experiences of care, adversely affected patients’ perceptions of orthodox healthcare.^30^

#### Barriers to SDM for MSK pain consultation and management (HCPs’ perspective)

##### Awareness, use and practice of SDM

HCP training was suggested to be related to the level of awareness and use of SDM. Only two studies reported the level of training (certified rheumatologist^28^) and professional experience of HCPs as 6–27 y.^28,30^ The age and gender of HCPs were not reported, except by Colmenares-Roa et al.^28^, who referenced a 1:1 male:female HCP ratio in their study. Middle-aged but highly skilled HCPs were more likely to be aware and incorporate SDM into their practice, while older HCPs were less likely to be familiar with the SDM concept.

##### Operationalization of SDM

Across care settings in LMICs, intention to use SDM within consultations was often limited by infrastructure, resources and practices (operational barriers). Studies were of moderate methodological quality. Subthemes and factors associated with infrastructure and resource barriers included consultation time, HCP workload and physical infrastructure.

Reduced consultation time negatively affected SDM.^28,30–32^ The degree of impact appears dependent on care settings and the nature of the MSK problem. For example, HCPs were more likely to perceive consultations for complex conditions such as fibromyalgia as less standardized, requiring greater investigations and longer explanation time.^28^

Reduced consultation time was also attributed to increased patient caseloads and subsequently higher demand on HCPs.^28,31,32^ High HCP workload was found to hamper the practice of SDM. The large numbers of patients seen by HCPs during the working day implies that consultation becomes oriented towards maximizing patient flow and monetary gain rather than patient needs. This was especially evident in publically funded care settings.^28,32^ For example, Gasparik et al.^32^ reported that the average consultation lasted 3.6 min, during which patients report symptoms in <1 min and the doctors, on average, ask four questions while patients ask less than one.

Constraints of the physical environment for consultation negatively impacted the doctor–patient relationship and SDM. Given the psychosocial impact of chronic MSK conditions on patients, crowded or open consultation spaces (with little/no privacy) made disclosure and discussion of patient’s emotional concerns very difficult.^28^ Subthemes associated with practice (operational) barriers included non-standardized or unclear treatment pathways, limited treatment choices, financial gain/incentives, HCPs’ impression of patients, health literacy, cultural factors and care models.

HCPs cited poor access to and knowledge of current evidence on aetiology and management, and there was an obvious lack of reference to locally relevant, evidence-based clinical guidelines for MSK pain management.^28,29,31^ Once a diagnosis was made, there was often no clear pathway for tailored and effective treatment plans in many care settings.^28,29,31^

Locally available treatment options were few, limiting treatment choices and mutualistic communication about ‘best treatment options’, in favour of a paternalistic care model.^28,29,31^ Where an agreed treatment pathway existed, patient management was by routine, and rarely individualized. In some settings, HCPs cited that the available management protocol is standardized and not amenable to tailoring for individual patients.^29,31^

HCPs were pressured to prescribe medications based on price rather than the best treatment option for individual patients.^28,31^ If a doctor already had a particular medication in mind before the consultation started (e.g. some physicians sign contracts with pharmaceutical companies to compensate for ‘low pay’), it was unlikely SDM would be practised at all, leading to paternalistic consultations that reduced patient involvement in their own care.^31^ Some physicians reported that colleagues who acted non-ethically were likely to make more money while those whose practices were more evidence-based were not likely to be noticed or rewarded for good practices,^31^ providing no motivation for patient-centred care.

HCPs’ minimization and labelling/categorization of certain patients (e.g. ‘patients with dependent and obsessed personalities’) in relation to their condition limited SDM. HCPs sometimes believed that patients’ problems resulted from their mind-set, medication dependency or attention seeking.^28,31^ Minimization and patient categorization led to mismatches between patient and doctor expectations, leading to frustration for both parties. Physicians were often found to describe patients as having ‘difficult’ traits, possibly due to unclear aetiology of the condition and complexities of managing psychological comorbidities. They became reluctant to see such patients or grew aggressive towards them.^28^

Additionally, HCPs felt patients’ lack of knowledge of their health conditions and management options would hinder SDM and patient-centred care.^31^ Even where HCPs expressed awareness of SDM, they were less inclined to incorporate it into management consultations. HCPs argued patients were not equipped to be involved in decision making and unlikely to want the responsibility for their own healthcare decision making.^31^ This was further compounded since many patients in these settings preferred their physicians to be the decision makers.^28–32^

HCPs’ were aware of patients’ lack of comprehension of health information. Subsequently HCPs’ perception of patients’ apparent lack of knowledge or ability to find and understand health-related information were found to limit the use of SDM.^28,31,32,34^ Patients’ health literacy was related to socio-economic status, determined by whether care was sought and/or provided in public or private settings.^28^ It was therefore possible that HCPs engaged differently with MSK patients depending on their perceived health literacy and healthcare setting (private vs public).

The healthcare model was a cross-cutting theme across the two analytical themes, descriptive themes and subthemes identified in this review. Models of care in included studies were mainly paternalistic (n=4)^28,29,31,32^, consumeristic (n=1)^29^ or unclassified (n=2).^33,34^

Within the paternalistic models, patients are expected to accept and comply with treatment instructions as given. HCPs are assumed to be the custodians of healthcare decision making, with little or no recourse to patient participation in management decisions, as exemplified by an orthopaedic surgeon: ‘*Patients choosing? That’s a concept I don’t manage very well. Patients don’t choose...*’.^29^ Evidently unsettled with the idea of patient choice, other HCPs in these settings appeared to attribute cultural dispositions as justification for paternalism, alluding to patients’ lack of familiarity with their own rights as a major barrier to SDM.^31^

The consumeristic model was more likely to be in a private healthcare setting. It appears to be service oriented, and patients come in-to consultations with expectations and demands, such as HCP manners, additional explanations and medical investigations that ‘must’ be provided. While a higher level of closeness and trust is often perceived by patients in this model, SDM is not necessarily being practised. For instance, an HCP may simply agree to or order more investigative procedures in an attempt to satisfy a patient’s expectations or create an impression of competence, or simply for financial gain.^30^

### Facilitators of SDM for MSK pain consultations

There was limited evidence for facilitators of SDM for MSK pain management in these settings due to the predominantly paternalistic models of care. However, included studies suggested training in SDM^28–34^ and decision aids as important facilitators.^28^ Specifically, HCPs with an awareness of SDM^23^ highlighted that the incorporation of SDM into the medical school curriculum and clinical training modelled through experienced practitioners may enhance the use of SDM in MSK pain management. Only one study explored patient decision aids for MSK pain consultations and suggested the need to involve patients in the development process such that decision aids are culturally acceptable.^28^

**Figure 3:**
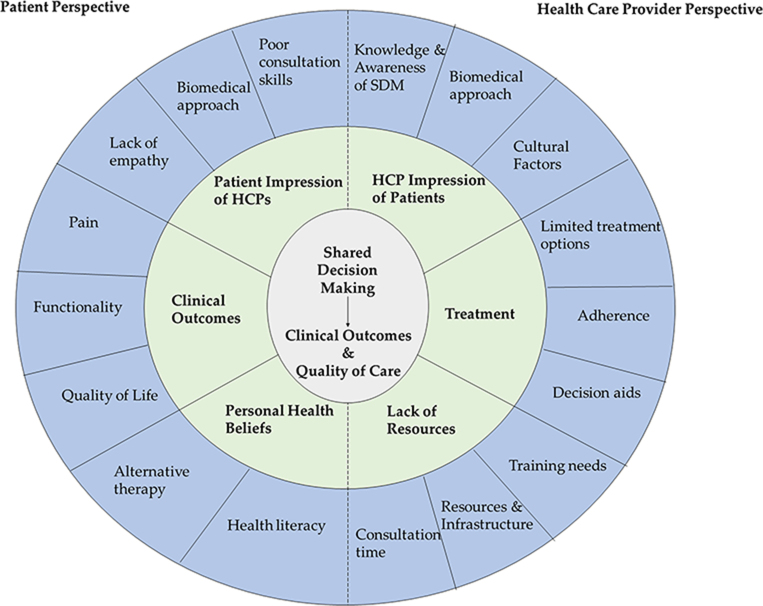
Conceptual model and summary of factors affecting shared decision making and MSK pain in LMICs.

### Summary and overall quality of the evidence

Overall, the review finds moderate evidence for a low level of awareness, use and practice of SDM in MSK consultations in LMICs. Figure 3 illustrates a conceptual model and summary of emerging themes in the review. Due to high consistency and the amount of data supporting the evidence for operationalization barriers to SDM across studies, it is highly likely that the review finding is a reasonable representation of factors affecting SDM from both the patient and HCP perspectives. Evidence supporting training and patient decision aids as facilitators of SDM for MSK pain management could not be substantiated by this review mainly due to a gap in the evidence base. Similarly, the association between SDM in MSK pain consultations and patient-oriented outcomes like pain, function and QOL was not clear (very low confidence).

## Discussion

There was generally a low level of awareness and practice of SDM for MSK pain management in LMICs. As a result, poor doctor–patient relationships were associated with inadequate treatment adherence and poor health-related outcomes from both the patient and HCP perspectives. Studies demonstrated evidence suggesting a discrepancy between HCP preparedness and patient expectations. The themes and subthemes identified in this review are interdependent (Figure 3). Unsatisfactory HCP–patient relationships further accentuate the burden of diseases and pressure on care systems, poor outcomes of care and subsequent interaction with the non-effective folk sector.^16,35^

This is the first systematic review regarding SDM for MSK pain in LMICs. Findings from this review regarding barriers (e.g. time constraints and perceived lack of applicability to patient ‘types’) and facilitators (e.g. decision tools, SDM training) of SDM in patient management are consistent with those of previous systematic reviews^20,36^ with data from HICs. However, we found that conceptualization of SDM and elements of it may have different values for HCPs and patients in LMICs. It is also possible, as seen from our findings, that, compared with HICs, concepts like responsibility for decision making (mostly projected to HCPs) are very different in LMICs. This study brings to light the current state of practice in MSK consultations with regards to SDM, but there is no evidence of high-quality research on theories that may underpin the awareness, use and practice of SDM in these settings. Hence it is difficult to map existing context-specific evidence implementation into practice in these settings and our findings must be interpreted with caution.

Notably, studies involved mostly physicians, and demonstrated poor multidisciplinary involvement in the care and research of MSK conditions in LMICs. This is in contrast to HICs, where multidisciplinary involvement is regarded as good practice and physiotherapists are at the forefront of care for MSK conditions.^6^ It has been suggested that, regardless of the presenting complaints or diagnosis, patients prefer to consult with doctors rather than other registered HCPs due to often misplaced health beliefs.^37^ Furthermore, existing care models and training in LMICs have made physicians less confident in the capability of other specialized HCPs and they are therefore less likely to refer patients who may have benefit from early and appropriate referral.^31^ Evidently both patient education and HCPs’ understanding of multidisciplinary teams are needed.

In many LMICs, credible patient appointment systems are missing and HCPs may see up to 90 patients per day.^38^ Thus paternalistic approaches to management, perceived as ‘simpler and faster’ methods of handling patient care, are natural options, resulting in compromised quality of care.^16^ Furthermore, patient involvement in SDM may be difficult in areas where patients are less likely to advocate for their own health due to cultural beliefs.^38^ Despite the growing reach of the internet, access, understanding scientific literature and language barriers limit the potential of patients in LMICs to access information regarding their health and reinforce the need for patients to continue deferring decision making to their HCPs. To achieve the WHO Millennium (and Sustainable) Development Goals, there needs to be greater emphasis on patient empowerment and health literacy in LMICs.

### Limitations

Given the dearth of relevant literature, there might be other factors that were not identified that affect SDM in the consultation and management of MSK pain conditions in LMICs. Moreover, it would have been difficult to establish that clinical outcomes for patients with MSK pain conditions in this review would be different if they had been involved in SDM, especially as the included randomized clinical trial was ongoing and is yet to present data on clinical outcomes. The cross-sectional studies also did not explicitly measure or report associations between SDM and clinical outcomes. Another limitation is that included studies did not cover all possible MSK pain conditions, thus the generalizability of the findings to all MSK pain conditions is difficult. Finally, statistical pooling of results (meta-analysis) was not possible due to a lack of quantitative studies and trial data.

### Implications for future research and policy

The burden of MSK pain conditions is a recognized public health issue of global health and socio-economic importance, especially in LMICs. Fortunately SDM could be a relatively simple and effective intervention^40^ in low-resource settings and for chronic conditions such as MSK pain. The current gap in the knowledge base as shown by this systematic review highlights the need for further research into the provision of care for MSK pain in these settings. It may also be an indicator of potential research obstacles (e.g. limited or no funding to conduct research in the field of MSK pain) in LMICs.

The findings of this review may be a springboard for more research into mitigating associated barriers and exploiting facilitators of quality care such as SDM training/awareness, patient education on self-management strategies, cultural appropriation of patient decision aids and health literacy in LMICs. Future studies of SDM for MSK pain management in LMICs will need to focus on methodologically robust, high-quality trials of non-pharmacological treatments, including exercises, psychological treatments and self-management support.

It is important to note that the current review synthesized evidence mainly from observational studies of pharmacological management of MSK pain despite established recommendations for the prudent use of medication and a focus on non-pharmacological treatment, including exercises, psychological treatments and self-management support.^6^ Furthermore, there was no mention of any locally adapted clinical guideline or of the WHO analgesic ladder, which are key for pain control.^41^ This highlights the lack of clinical standards and established and agreed protocols that could be used to tailor clear treatment plans for patients in these settings. There is an urgent need for discussions broaching MSK pain management and viable treatment options that may be locally available to patients in LMICs.

As with previous reviews of studies of SDM in HICs,^17,19,21^ the current review is unable to comment on the overall effectiveness of SDM regarding patient-related health outcomes. This is partly due to non-use of standardized and validated measures for SDM in clinical practice, as well as the wide variations in the components, contents and practice of SDM across condition-specific and healthcare settings. A good starting point could be a consensus work on what good practice should be and the use of validated measures such as that developed by Elwyn et al.^42^ In addition, it is important to note that SDM may not necessarily solve all the problems associated with inefficient management of MSK patients. A careful consideration of under what circumstances, when and how SDM might be carefully incorporated within local contexts is important. Barriers to SDM as expressed by patients and HCPs also need to be addressed from local and context-specific perspectives in order to mitigate possible unintended or undesirable effects of SDM in LMICs.

## Conclusions

A poor level of awareness, use and practice of SDM appeared to lead to an ineffective doctor–patient relationship, poor adherence to recommended treatments and poor self-management strategies among patients with MSK pain in LMICs. Quality care incorporating SDM is still a forgotten dimension of MSK care in LMICs. Promotion of health literacy initiatives and mutualistic care models to both HCPs and patients is likely to reduce physician workload, improve adherence to prescribed treatments and increase patient outcomes and overall quality of care for MSK pain patients in LMICs.

## Appendix 1. OVID MEDLINE search strategy

Key: / —an index term; exp—before an index term indicates all subheadings selected; ?—optional character; .mp—search in ‘multiple places’, including title, abstract, subject heading, keyword; ti,ab,kw.—search in title/abstract/keyword; $(*n*)—at the end of a term indicates that this term has been truncated (by *n* characters); adj(*n*)—search for two terms that appear adjacent to, or appear within *n* words of, each another.

**Table:**

1	exp pain/or (pain$ or ache$1 or aching or stiff or stiffness or impingement$ or strain$ or sprain$).ti,ab,kw.
2	exp Musculoskeletal System/or exp Lower extremity/or exp Upper extremity/or exp back/or neck/or (muscle$ or tendon$ or ligament$ or knee or knees or hip$ or ankle$ or foot or feet or shoulder$ or elbow$1 or wrist$1 or hand$1 or arm$1 or forearm$1 or back or neck or spine or spinal or lumbar or cervical or joint$ or rotator cuff).ti,ab,kw.
4	exp Musculoskeletal Diseases/or exp Musculoskeletal pain/or exp Back Pain/or Neck pain/or rheumatology/or exp “Sprains and Strains”/or anterior cruciate ligament injuries/or iliotibial band syndrome/or medial tibial stress syndrome/or tibial meniscus injuries/or Whiplash Injuries/or rotator cuff injuries/or (musculoskeletal or MSK or arthriti$ or bursitis or capsulitis or tendonitis or tendinitis or tendinopath$ or tenosynovitis or ((chronic or multisite or “multi site”) adj3 pain) or pain syndrome$ or osteoarthr$ or arthralgia$ or arthrosis or rheumat$ or (joint$ adj3 disease$) or myositis or myopath$ or myalgia$ or whiplash$ or impingement syndrome$).ti,ab,kw
4	(1 and 2) or 3
5	((share$ or sharing or informed) and (decision$ or deciding or choice$)).ti. or ((share$ or sharing or informed) adj decision$) or informed choice$.ab,kw. or decision aid$.ti,ab,kw.
6	Decision Making/or Decision Support Techniques/or Decision Support Systems, Clinical/or Choice Behavior/or ((decision$ or choice$) and (making or support$ or behavio?r$)).ti. or (decision making or decision support$ choice behavio?r$.ab,kw.
7	Patient Participation/or ((patient$ or consumer$ or client$) and (involvement or involving or participation or participating)).ti. or ((patient or consumer or client) adj (participation or involvement)).ab,kw. or ((patient$ or person$ or client$ or consumer$) adj (centred or centered or focused or oriented)).ti,ab,kw.
8	exp Professional-Patient Relations/or exp Nurses/or exp Physicians/or (nurse$ or physician$ or clinician$ or doctor$ or general practitioner$ or GP or GPs ((healthcare or healthcare) adj (professional$ or provider$)) or resident or residents).ti.
9	exp Patients/or (patient$ or consumer$ or people$ or client$).ti.
10	5 or (6 and 7) or (6 and 8 and 9) or (7 and 8 and 9)
11	Developing Countries/or asia/or asia, central/or asia, northern/or asia, southeastern/or asia, western/or middle east/or far east/or africa/or africa, northern/or “africa south of the sahara”/or africa, central/or africa, eastern/or africa, southern/or africa, western/or caribbean region/or west indies/or central america/or latin america/or South America/or Europe, Eastern/
12	((middle income$ or developing or under developed or underdeveloped or 3rd world or third world or LAMI or LMIC or poor income or low$ income or less$ developed or least developed) adj (countr$ or nation or nations or econom$)) or (South or Southern or South East or Southeast$ or West$ or Middle or Far) adj Asia$) or Africa$ or ((Latin or Central or South) adj America$) or Caribbean$ or West Indies or East$ Europe$).ti,ab,kw or ((Afghan$ or Angola$ or Armenia$ or Bangladesh$ or Benin$ or Bhutan$ or Bolivia$ or Burkina Faso$ or Upper Volta$ or Burundi$ or Cabo Verd$ or Cape Verd$ or Cambodia$ or Cameroon$ or Central African Republic$ or Chad$ or Comoros or Comorian or Congo$ or Cote d’Ivoire or Ivory Coast or Ivorian$ or Djibouti$ or Egypt$ or El Salvador$ or Eritrea$ or Ethiopia$ or Gambia$ or Georgia$ or Ghana$ or Guatemala$ or Guinea$ or Haiti$ or Hondura$ or India$ or Indonesia$ or Jordan$ or Kenya$ or Kiribati$ or Korea$ or Lesotho or Mosotho or Basotho or Liberia$ or Madagasca$ or Malagasy or Kosov$ or Kyrgyz$ or Lao$ or Malawi$ or Mali or Mali’s or Malian$ or Mauritania$ or Micronesia$ or Moldov$ or Mongolia$ or Morocc$ or Mozambi$ or Myanmar$ or Burma$ or Burmese$ or Nepal$ or Nicaragua$ or Niger$ or Pakistan$ or PNG$ or Philippines$ or Filipino$ or Rwanda$ or Sao Tome$ or Principe$ or Senegal$ or Sierra Leone$ or Solomon Island$ or Somalia$ or Sudan$ or Sri Lanka$ or Swaziland$ or Syria$ or Tajik$ or Tanzania$ or Timor$ or Togo$ or Tunisia$ or Uganda$ or Ukrain$ or Uzbek$ or Vanuatu$ or Vietnam$ or West Bank$ or Gaza$ or Palestin$ or Yemen$ or Zambia$ or Zimbabwe$ or Albania$ or Algeria$ or Argentin$ or Samoa$ or Azerbaijan$ or Belarus$ or Belize$ or Bosnia$ or Herzegovia$ or Botswana$ or Brazil$ or Bulgaria$ or China$ or Chinese$ or Colombia$ or Costa Rica$ or Croatia$ or Cuba$ or Dominica$ or Ecuador$ or Fiji$ or Gabon$ or Grenad$ or Guyan$ or Iran$ or Iraq$ or Jamaica$ or Kazakh$ or Leban$ or Libya$ or Macedonia$ or Malaysia$ or Maldiv$ or Marshall Island$ or Mauriti$ or Mexic$ or Montenegr$ or Namibia$ or Nauru$ or Panama$ or Paraguay$ or Peru$ or Romania$ or Russia$ or Serbia$ or South Africa$ or Suriname$ or Lucia$ or Vincent$ or Thai$ or Tonga$ or Turk$ or Turkmen$ or Tuvalu$ or Venezuela$).mp
13	4 and 10 and (11 or 12)

### Box 1. Eligibility criteria

**Table:**

**Notes:** The PICO format will be used to screen across the inclusion criteria sequentially, in the following order: Study design yes/no; Participants yes/no; Intervention yes/no; Outcomes yes/no; Setting yes/no; FINAL DECISION yes/no. A NO at any stage in the process leads to automatic exclusion of the article		
	Inclusion criteria	Exclusion criteria
Study design	Primary or secondary care consultations in LMICs Any study design—RCT, non-RCT: before and after study, cross-sectional study and qualitative study	Single case reports Narrative reviews, letters, editorials, commentaries and meeting abstracts without data Biomechanical, lab studies, animal studies Non-peer-reviewed articles, e.g. government gazettes
Participants and conditions of interest	Adults ≥18 y of age diagnosed with MSK pain or symptoms. Consider including studies where the MSK pain is discussed (including symptomatic or diagnostic labels)	Children Patients without MSK conditions or pain MSK pain with red flag diagnoses, e.g. suspected fractures, cancer, inflammatory arthritis, crystal disease, spondyloarthropathy, polymyalgia rheumatica, pregnancy-related pain problems, urgent cases (e.g. cauda equina syndrome) or vulnerable patients (e.g. recent trauma, cognitive impairment, dementia, terminal illness) Studies in specific subpopulations (e.g. patients with severe mental illness)
Interventions or exposures	Discussion of potential/current management options with an HCP	No interventions or discussion of management options
Comparisons or control groups	Any, general population	
Outcomes of interest	Primary outcomes: awareness, knowledge and health behaviours regarding SDM by HCPs and/or patients; factors affecting SDM (facilitators and barriers) in those settings Secondary outcomes: evidence of the effect of SDM on clinical outcomes such as pain, functional decline, quality of life	
Setting	Countries classified by the World Bank as LMICs at any point within the last 5 y	Studies based on individuals living in HICs according to World Bank classification

PICO: patient/problem, intervention of interest, comparison, outcome.
